# SLIC Superpixel-Based *l*_2,1_-Norm Robust Principal Component Analysis for Hyperspectral Image Classification

**DOI:** 10.3390/s19030479

**Published:** 2019-01-24

**Authors:** Baokai Zu, Kewen Xia, Tiejun Li, Ziping He, Yafang Li, Jingzhong Hou, Wei Du

**Affiliations:** 1 School of Electronics and Information Engineering, Hebei University of Technology, Tianjin 300401, China; zubaokai@163.com (B.Z.); heziping1102@126.com (Z.H.); houjingzhong2002@163.com (J.H.); 2 School of Mechanical Engineering, Hebei University of Technology, Tianjin 300401, China; 1993076@hebut.edu.cn; 3 Faculty of Information Technology, Beijing University of Technology, Beijing 100124, China; yafangli@bjut.edu.cn; 4 College of Resources and Environment, Huazhong Agricultural University, Wuhan 430070, China; weidu@webmail.hzau.edu.cn

**Keywords:** Hyperspectral Image, Robust Principal Component Analysis (RPCA), Simple Linear Iterative Clustering (SLIC), superpixel segmentation

## Abstract

Hyperspectral Images (HSIs) contain enriched information due to the presence of various bands, which have gained attention for the past few decades. However, explosive growth in HSIs’ scale and dimensions causes “Curse of dimensionality” and “Hughes phenomenon”. Dimensionality reduction has become an important means to overcome the “Curse of dimensionality”. In hyperspectral images, labeled samples are more difficult to collect because they require many labor and material resources. Semi-supervised dimensionality reduction is very important in mining high-dimensional data due to the lack of costly-labeled samples. The promotion of the supervised dimensionality reduction method to the semi-supervised method is mostly done by graph, which is a powerful tool for characterizing data relationships and manifold exploration. To take advantage of the spatial information of data, we put forward a novel graph construction method for semi-supervised learning, called SLIC Superpixel-based $l_{2,1}$-norm Robust Principal Component Analysis (SURPCA_2,1_), which integrates superpixel segmentation method Simple Linear Iterative Clustering (SLIC) into Low-rank Decomposition. First, the SLIC algorithm is adopted to obtain the spatial homogeneous regions of HSI. Then, the $l_{2,1}$-norm RPCA is exploited in each superpixel area, which captures the global information of homogeneous regions and preserves spectral subspace segmentation of HSIs very well. Therefore, we have explored the spatial and spectral information of hyperspectral image simultaneously by combining superpixel segmentation with RPCA. Finally, a semi-supervised dimensionality reduction framework based on SURPCA_2,1_ graph is used for feature extraction task. Extensive experiments on multiple HSIs showed that the proposed spectral-spatial SURPCA_2,1_ is always comparable to other compared graphs with few labeled samples.

## 1. Introduction

Hyperspectral Images (HSIs) provide comprehensive spectral information of the materials’ physical properties [1], which is applied in ecosystem monitoring [2], agricultural monitoring [3,4], environmental monitoring [5], forestry [6], urban growth analysis [7,8], and mineral identification [9,10]. The abundant hyperspectral image bands bring rich spectral information, but they result in the “Hughes phenomenon” [11,12] that reduces the accuracy and efficiency of the classification task. By reducing the dimensionality of the data, more compact low-dimensional hyperspectral images can be obtained. Therefore, it is important and practically significant to perform appropriate dimensionality reduction on hyperspectral images.

Dimensionality reduction refers to finding the low-dimensional representation of the original data by eliminating the redundant elements and retaining their main features, which can overcome the “Curse of dimensionality” problem. Dimensionality reduction mainly includes feature selection and feature extraction [13]. Feature selection methods reduce the dimensions of the original data by selecting the most representative and distinguishing features [14]. Feature extraction methods combine multiple features linearly and non-linearly on the basis of maintaining the data structure information [15]. Recently, many dimensionality reduction algorithms have been put forward. The most classic ones are the early Principal Component Analysis (PCA) [16], Linear Discriminant Analysis (LDA) [17], and Independent Component Analysis (ICA) [18,19,20]. In recent years, feature extraction has quickly become one of the hotspots in machine learning and data mining. Compared with the traditional dimensionality reduction method, the main difference of manifold learning is its ability to maintain the invariance of the data structure. The most representative algorithms include Laplacian Eigenmap [21], Isomap [22], and Local Linear Embedding (LLE) [23]. A multimetric learning approach that combines feature extraction and active learning (AL) is proposed to deal with the high dimensionality of the input data and the limited number of the labeled samples simultaneously [24]. To simultaneously deal with the two issues mentioned above, a regularized multimetric active learning (AL) framework is proposed [25].

With the rapid development of data acquisition and storage technology, many unlabeled samples are available, but labeled samples are more difficult to collect. If the training samples are limited, the machine learning methods may be confronted with over-fitting problems when dealing with high-dimensional small sample size problems [26,27]. For the classification of hyperspectral images (HSIs), good classification results usually require many labeled samples. To resolve the over-fitting problem, semi-supervised learning was proposed to utilize both labeled samples and the unlabeled samples that conveyed the marginal distribution information to enhance the algorithmic performance [28,29,30]. Semi-supervised dimensionality reduction combines semi-supervised learning with dimensionality reduction, and has gradually become a new branch in the machine learning field. Semi-supervised dimensionality reduction methods not only use the supervised information of the data, but also maintain the structural information of the data. In the dimensionality reduction method, the promotion of the supervised dimensionality reduction method to a semi-supervised method is mostly done by graph-based methods. Therefore, graph-based semi-supervised learning methods have gained a great deal of attention, which construct adjacency graph to extract the local geometry of the data. Graph is a powerful data analysis tool, which is widely used in many research domains, such as dimension reduction [21,22,23], semi-supervised learning [31,32], manifold embedding, and machine learning [33,34]. In graph-based methods, each point is mapped to a low-dimensional feature vector, trying to maintain the connection between vertices [30]. The procedure of graph construction effectively determines the potential of the graph-based learning algorithms. Thus, for a specific task, a graph which models the data structure aptly will correspondingly achieve a good performance. Therefore, how to construct a good graph has been widely studied in recent years, and it is still an open problem [35].

Within the last several years, numerous algorithms for feature extraction have been put forward. PCA is a widely-used linear subspace algorithm that seeks a low-dimensional representation of high-dimensional data. PCA works against small Gaussian noise in data effectively, but it is highly sensitive to sparse errors of high magnitude. To solve this problem, Candès [36] and Wright et al. [37] proposed Robust Principal Component Analysis (RPCA), which intends to decompose the observed data into a low-rank matrix and a sparse noises matrix. In [38], a novel feature extraction method based on non-convex robust principal component analysis (NRPCA) was proposed for hyperspectral image classification. Nie et al. developed a novel model named graph-regularized tensor robust principal component analysis (GTRPCA) for denoising HSIs [39]. Chen et al. denoised hyperspectral images using principal component analysis and block-matching 4D filtering [40].

The above mentioned methods suggest that the pixels in a hyperspectral image lie in a low-rank manifold. However, the spatial correlation among pixels is not revealed. For HSIs, the adjacent pixels’ correlation is typically extremely high [41], which has potential low-rank attributes. Pixels in homogenous areas usually consist of similar materials, whose spectral properties are highly similar and can be taken as being approximately in the same subspace. By extracting the low-rank matrix of the data, the low-dimensional structure of each pixel is revealed and we can classify each pixel more accurately. Xu et al. put forward a low-rank decomposition spectral-spatial algorithm, which incorporates global and local correlation [42]. Fan et al. integrated superpixel segmentation (SS) into Low-rank Representation (LRR) and proposed a novel denoising method called SS-LRR [43]. A multi-scale superpixel based sparse representation (MSSR) for HSIs’ classification is proposed to overcome the disadvantages of utilizing structural information [44]. Sun et al. presented a novel noise reduction method based on superpixel-based low-rank representation for hyperspectral image [45]. In [46], Mei et al. proposed a new unmixing method with superpixel segmentation and LRR based on RGBM. Tong et al. proposed multiscale union regions adaptive sparse representation (MURASR) by multiscale patches and superpixels [47]. A novel method, robust regularization block low-rank discriminant analysis, is proposed for HSIs’ feature extraction [48].

**Figure 1:**
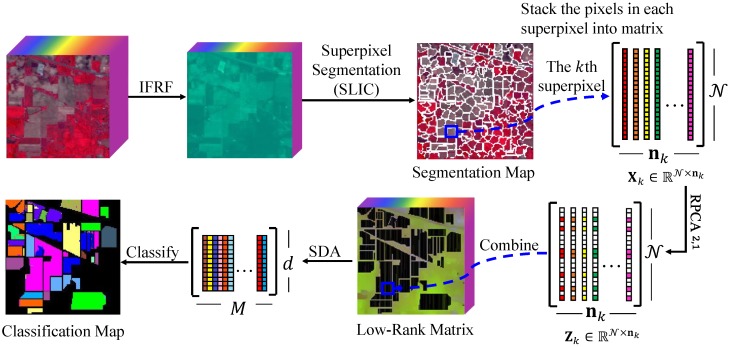
Formulation of the proposed SLIC superpixel-based $l_{2,1}$-norm robust principal component analysis for HSIs classification.

Spatial information can play a very important role in mapping data. However, many graph construction methods do not make full use of data’s spatial information. For example, in hyperspectral images, adjacent pixels in a homogenous area usually belong to the same category. Therefore, how to consider the features of the actual data globally to further improve the semi-supervised dimensionality reduction performance is one of the main research components of this paper. Considering the spatial correlative and spectral low-rank characteristics of pixels in HSIs, we integrate the Simple Linear Iterative Clustering (SLIC) segmentation method into low-rank decomposition. Figure 1 shows the formulation of the proposed SLIC Superpixel-based $l_{2,1}$-norm Robust Principal Component Analysis (SURPCA_2,1_) for hyperspectral image classification. As shown in Figure 1, we preprocess the hyperspectral image by the Image Fusion and Recursive Filtering feature (IFRF), which eradicates the noise and redundant information concurrently [12]. The proposed method divide the image into multiple homogeneous regions by the superpixel segmentation algorithm SLIC. The pixels in each homogeneous region may belong to the same ground object category. Therefore, we stack the pixels in the same homogeneous region into a matrix. Due to the low-rank property of the matrix, RPCA is used to recover the low-dimensional structure of all pixels in the homogeneous region. Then, we combine these low-rank matrices together to an integrated low-rank graph. In addition, we process the semi-supervised discriminant analysis for dimension reduction, which takes advantage of the labeled samples and the distribution of the whole samples. The *k*-Nearest Neighbor (*k*NN) algorithm is applied to handle the low-rank graph for the regularized graph of semi-supervised discriminant analysis. Finally, we implement the Nearest Neighbor classifier method. We summarize the main contributions of the paper in the following paragraphs.

- Inspired by robust principal component analysis and superpixel segmentation, we put forward a novel graph construction method, SLIC superpixel-based $l_{2,1}$-norm robust principal component analysis. The superpixel-based $l_{2,1}$-norm RPCA extracts the low-rank spectral structure of all pixels in each uniform region, respectively.
- The simple linear iterative clustering addresses the spatial characteristics of hyperspectral images. Consequently, the SURPCA_2,1_ graph model can classify pixels more accurately.
- To investigate the performance of the SLIC superpixel-based $l_{2,1}$-norm robust principal component analysis graph model, we conducted extensive experiments on several real multi-class hyperspectral images.

We start with a scientific background in Section 2. Section 3 deciphers the HSIs classification with $l_{2,1}$-norm robust principal component analysis. Section 4 performs comparative experiments on real-world HSIs to examine the performance of the proposed graph. We provide the discussion in Section 5. Conclusions are presented in Section 6.

## 2. Scientific Background

### 2.1. Graph-Based Semi-Supervised Dimensionality Reduction Method

In the dimensionality reduction method, the promotion of supervised method to semi-supervised method is mostly done by graph-based methods. Maier et al. [49] showed that different graph structures obtain different results in the same clustering algorithm. A good graph construction method has great influence in graph-based semi-supervised learning. Therefore, how to construct a good graph sometimes seems to be more important than a good objective function [50].

Graph is composed by nodes and edges, which represents the structure of the entire dataset. The nodes represent the sample points, and the edges’ weight represents the similarity between the nodes. Generally, the greater the weight is, the higher the similarity between the nodes will be. If the edge does not exist, the similarity between the two points will be zero. The similarity between nodes is usually measured by distance, such as Euclid, Mahalanobis, and Chebyshev distance [51]. Different graph construction methods have great impact on the performance of classification results. The graph construction process mainly includes the graph structure and the edges’ weight function. Two commonly used graph structures are Fully Connected graphs and Nearest Neighbor graphs (*k*-nearest neighbor graph and ε-ball Graph).

- Fully Connected graphs [21,52]: In the fully connected graph, all nodes are connected by edges whose weights are not zero. The fully connected graphs are easy to construct and have good performance for semi-supervised learning methods. However, the disadvantage of the fully connected graphs is that it requires processing all nodes, which would lead to high computational complexity.
- *k*-nearest neighbor graph [21]: Each node in the *k*-nearest neighbor graph is only connected with *k* neighbor in a certain distance. The samples $x_{i}$ and $x_{j}$ are considered as neighbor if $x_{i}$ is among the *k* nearest neighbor of $x_{j}$ or $x_{j}$ is among the *k*-nearest neighbor of $x_{i}$.
- ε-ball graph [21]: In the ε-ball graph, the connections between data points occur in the neighborhood of radius ε. That is to say, if the distance between $x_{i}$ and $x_{j}$ exists $d(x_{i},x_{j})\leq \varepsilon ,\varepsilon \in R,\varepsilon >0$, there will be a neighbor relationship between these two nodes. Therefore, the connectivity of the graph is largely influenced by the parameter ε.

It is also necessary to assign weight for the connected edges W. The followings are commonly used weight functions:

- Inverse of Euclidean distance [53],
  (1) $$W_{ij}=\left\{\begin{array}{cc} {∥x_{i}-x_{j}∥}^{-2}, & if\;x_{i}\in N_{k}\left(x_{j}\right)\;orx_{j}\in N_{k}\left(x_{i}\right)\\ 0, & otherwise. \end{array}\right.$$
- 0–1 weighting [21],
  (2) $$W_{ij}=\left\{\begin{array}{cc} 1, & if\;x_{i}\in N_{k}\left(x_{j}\right)\;orx_{j}\in N_{k}\left(x_{i}\right)\\ 0, & otherwise. \end{array}\right.$$
- Heat kernel weighting [21],
  (3) $$W_{ij}=\left\{\begin{array}{cc} \exp\left(-\frac{{∥x_{i}-x_{j}∥}^{2}}{2\sigma^{2}}\right), & if\;x_{i}\in N_{k}\left(x_{j}\right)orx_{j}\in N_{k}\left(x_{i}\right)\\ 0, & otherwise. \end{array}\right.$$

*k*NN graph can make full use of the local information of adjacent nodes. Since *k*NN graph is sparse, it can solve the computational complexity and storage problem in the fully connected graph. The parameters *k* and ε depend on the data density, which is difficult to select. The choice of neighbor size parameter is the key to the effectiveness of the graph-based semi-supervised learning method. The Nearest Neighbor graph lacks global constraints of the data points, and its representation performance is greatly reduced when the data is seriously damaged [21].

### 2.2. Simple Linear Iterative Clustering

The superpixel is an image segmentation technique proposed by Xiaofeng Ren in 2003 [54]. It refers to the image regions with certain visual meanings composed of adjacent pixels which have similar physical characteristics such as texture, color, brightness, etc. [55]. The superpixel segmentation methods segment pixels and replace a large number pixels with a small number of superpixels, which greatly reduces the complexity of image post-processing. It has been widely used in computer vision applications such as image segmentation, pose estimation, target tracking, and target recognition. The boundary information is relatively obvious between the superpixels. Achanta et al. introduced a simple linear iterative clustering algorithm to efficiently produce superpixels that are compact and nearly uniform [56].

The superpixel algorithms are broadly classified into graph-based and gradient-ascent-based algorithms. In graph-based algorithms, each pixel is treated as a node in a graph, and edge weights between two nodes are set proportional to the similarity between the pixels. Superpixel segmentations are extracted by effectively minimizing a cost function defined on the graph [57]. Simple linear iterative clustering was proposed in 2010, and is simple to use and understand. Simple linear iterative clustering is based on the *k*-means clustering algorithm [58], which is done in the five-dimensional Labxy space, where Lab is the pixel color vector in CIELAB color space, which is widely considered as perceptually uniform for small color distances, and x,y is the pixel position [57]. Starting from an initial rough clustering, the clusters from the previous iteration are refined to obtain better segmentation in each iteration by gradient ascent method until convergence. Then, the distance metrics of the five-dimensional feature vectors are constructed. Finally, the pixels are clustered in the image locally [57,59]. The method has a high comprehensive evaluation index in terms of computational speed and superpixel shape, which achieves good segmentation results. It has two significant advantages compared with other algorithms. One is that it restricts the search space to proportionate the size of the superpixels, which can significantly reduce the number of distance calculations during the optimization process. The other is that the weighted distance combines color and spatial metrics while control the number and compactness of superpixels.

The SLIC method performs a *k*-means-based local clustering algorithm in a five-dimensional space distance measurement, which achieves compactness and regularity in superpixel shapes. *L* represents luminosity, and ranges from 0 (black) to 100 (white). The color-associated elements *a* and *b* represent the range of colors from magenta to green and yellow to blue, respectively. The Lab color model not only contains the entire color gamut in RGB and CMYK, but also colors they cannot present. The Lab space can express all colors perceived by the human eyes, and is perceptually uniform for small color distance. Instead of directly using the Euclidean distance in this five-dimensional space, SLIC introduces a new distance measure that considers superpixels’ size. For an image with *N* pixels, the SLIC algorithm takes a desired number of equally-sized superpixels N/K as input. There is a superpixel center at each grid interval $S=\sqrt{N/K}$. SLIC is easy in practice application because it only needs to set the unique superpixels’ desired number *K*.

**Figure 2:**
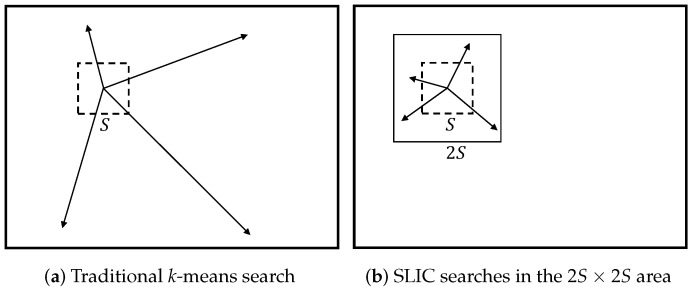
Simple linear iterative clustering (SLIC) reducing the search regions.

The clustering procedure begins with *K* initial cluster centers (seeds) $C_{k}={\left[l_{k},a_{k},b_{k},x_{k},y_{k}\right]}^{T}$, which are sampled on regular grids with an *S* pixels interval. Each seed is moved to the lowest gradient position in its 3×3 neighborhood to avoid centering the superpixel at the edge position and reduce the chance of seeding with noisy pixels. We assume that the cluster center is located within a 2S×2S area since the spatial extent of a superpixel is about $S^{2}$. This strategy can accelerate the convergence process, as shown in Figure 2. The SLIC superpixel segmentation algorithm is a simple local *k*-means algorithm, whose search area is the 2S×2S area nearest to each cluster center. Since each pixel is searched by multiple seed pixels, the cluster center of the pixel is the seed with the minimum value.

Let ${\left[l_{i},a_{i},b_{i},x_{i},y_{i}\right]}^{T}$ be the five-dimensional vector of a pixel. Calculate the distance between each pixel and the seeds. The distance between the pixel and seed $C_{k}$ is shown as follows:

(4) $$D_{lab}=\sqrt{{\left(l_{k}-l_{i}\right)}^{2}+(a_{k}-a_{i}{)}^{2}+(b_{k}-b_{i}{)}^{2}}$$

(5) $$D_{xy}=\sqrt{{\left(x_{k}-x_{i}\right)}^{2}+(y_{k}-y_{i}{)}^{2}}$$

(6) $$D_{S}=D_{lab}+\frac{l}{S}D_{xy}$$

The variable *l* controls the superpixels’ compactness. The larger the value *l* is, the more compact the cluster will be.

The above steps are repeated iteratively until convergence, which means that the cluster center of each pixel is no longer changing. *l* ranges from 1 to 20. Here, we chose l=10, which roughly matches the empirically perceptually meaningful Lab distance and provides good balance between spatial proximity and color similarity.

After the iterative optimization, superpixel multi-connected conditions may occur. Some superpixels’ sizes may be too small, a single superpixel may be segmented into a plurality of discontinuous superpixels, etc., which can be solved by enhancing the connectivity of the superpixels. The solution is to reassign discrete and small-size superpixels to their adjacent superpixels until all the pixels have been traversed.

### 2.3. Robust Principal Component Analysis

Decomposing the matrix into low-rank and sparse parts can separate the main components from outliers or noise, which is suitable for mining low-dimensional manifold structures in high-dimensional data. We stack the pixels in the same homogeneous region into a matrix $X_{k}\in R^{N\times n_{k}}$, where $n_{k}$ is the pixel number in the *k*th homogeneous region. Since the matrix has low-rank property, a low-rank matrix recovery algorithm is employed to recover the low-dimensional structure of all pixels in the homogeneous region. After recovering the low-rank matrix, the low-rank matrices are combined together into an integrated low-rank graph. The low-rank matrix restored by low-rank representation is a square matrix, while the dimensionality of the low-rank matrix restored by robust principal component analysis is the same as the original $X_{k}\in R^{N\times n_{k}}$. Since the number of pixels in each homogeneous region is different, robust principal component analysis is suitable for restoring the low-dimensional structures of all pixels in a homogeneous region here.

Principal Component Analysis is a fundamental operation in data analysis, which assumes that the data approximately lies in a low-dimensional linear subspace [16]. The success and popularity of PCA reveals the ubiquity of low-rank matrices. When the data are slightly damaged by small noise, it can be calculated stably and efficiently by singular value decomposition. However, noise and outliers limit PCA’s performance and applicability in real scenarios.

Suppose we stack samples as column vectors of a matrix $X\in R^{m\times n}$. The problem of classical PCA is to seek the best estimate of Z that minimizes the difference between Z and X:

(7) $$\begin{array}{c} \min_{Z,E}\;{∥E∥}_{F}\\ \;s.t.\;\;rank(Z)\leq r,X=Z+E \end{array}$$

where ${∥.∥}_{F}$ denotes the Frobenius norm. *r* is the upper-bound rank of the low-rank matrix Z. This problem can be efficiently solved via singular value decomposition (SVD) when the noise E is independent and identically distributed (i.i.d.) small Gaussian noise. A major disadvantage of classic PCA is its robustness to grossly corrupted or outlying observations [16]. In fact, even though only one element in the matrix changes, the obtained estimation matrix is far from the ground truth.

Recently, Wright et al. [37] proposed an idealized robust PCA model to extract low-rank structures from highly polluted data. In contrast to the traditional PCA, the proposed RPCA can exactly extract the low-rank matrix and the sparse error E (relative to Z). The robust principal component analysis model is expressed as follows:

(8) $$\begin{array}{c} \min_{Z,E}\;rank\left(Z\right)+\lambda {∥E∥}_{0}\\ \;s.t.\;X=Z+E \end{array}$$

where λ is to balance low-rank matrix Z and the error matrix E. We recover the pair $\left(Z_{0},E_{0}\right)$ that were generated from data X. Unfortunately, Equation (8) is a very non-convex optimization problem with no valid solution. Replacing the rank function with the kernel norm, and replacing the $l_{0}$-norm with the $l_{1}$-norm, results in the following relaxing Equation (8) convex surrogate:

(9) $$\begin{array}{c} \min_{Z,E}\;{∥Z∥}_{*}+\lambda {∥E∥}_{1}\\ \;s.t.\;X=Z+E \end{array}$$

The optimization in Equation (9) can be treated as a general convex optimization problem. Currently, the optimization algorithms of RPCA mainly contain Iterative Thresholding (IT), Accelerated Proximal Gradient (APG), and DUAL. However, the iterative thresholding algorithm proposed in [37] converges slowly. The APG algorithm is similar to the IT algorithm, but the number of iterations is significantly reduced. In addition, the DUAL algorithm does not require the complete singular value decomposition of the matrix, so it has better scalability than the APG algorithm. Recently, some researchers proposed Augmented Lagrangian Multiplier (ALM) algorithm, which has a faster speed than previous algorithms. The exact ALM (EALM) method turns out that it has a satisfactory *Q*-linear convergence speed, whereas the APG is theoretically only sub-linear. A slight improvement over the exact ALM leads to an inexact ALM (IALM) method, which converges as fast as the exact ALM. Therefore, the IALM method is applied to obtain the optimization here.

## 3. Methodology

In the above section, we discuss the simple linear iterative clustering and robust principle component analysis. Furthermore, labeled and unlabeled samples can be exploited simultaneously by depicting the underlying superpixels’ low-rank subspace structure. Considering this, we have attempted to decipher our present work the superpixel segmentation and $l_{2,1}$-norm pobust principal component analysis feature extraction model.

### 3.1. Superpixel Segmentation and $l_{2,1}$-Norm Robust Principal Component Analysis

Hyperspectral images preserve low-rank properties, but the category composition corresponding to a hyperspectral image is very complicated in practical applications. The robust principal component analysis model supposes that the data lie in one low-rank subspace, which makes it difficult to characterize the structure of the data with multiple subspaces. Hence, we explore the robust principal component analysis via superpixel, which is usually defined as a homogeneous region. Here, we employ the SLIC superpixel segmentation method to segment the hyperspectral image into spatially homogeneous regions. Each superpixel is a unit with consistent visual perception, and the pixels in one superpixel are almost identical in features and belong to the same class. Initially, we extract the information of the HSIs by image fusion and recursive filtering [12].

Suppose $R=\left(r_{1},r_{2},\cdots ,r_{D}\right)\in R^{M\times D}$ represent the original hyperspectral image, which has *M* pixels and *D*-dimensional bands. We segment the whole hyperspectral bands into multiple subsets spectrally. Each subset is composed of *L* contiguous bands. The number of subsets $N=\left\lfloor D/L\right\rfloor$, which represents the largest integer not greater than D/L. The *i*th (i∈(1,2,⋯,N)) subset is shown as follows:

(10) $$P^{i}=\left\{\begin{array}{c} \left(r_{L*(i-1)+1},\cdots ,r_{L*i}\right),\;if\;\left(L*i\right)\leq D\\ (r_{L*(i-1)+1},\cdots ,r_{D}),\;otherwise. \end{array}\right.$$

Afterwards, the adjacent bands of each subset are fused by an image fusion method (i.e., the averaging method). For example, the *i*th fusion band, that is, the image fusion feature $F_{i}$, is as follows:

(11) $$Q^{i}=\frac{\sum_{n=1}^{N_{i}}P_{n}^{i}}{N_{i}}$$

Here, $P_{n}^{i}$ refers to the *n*th band in the *i*th subset of a hyperspectral image. $N_{i}$ is the band number in the *i*th subset. After image fusion, we remove the noise pixels and redundant information for each subset. Then, we transform the domain recursive filtering algorithm on $Q^{i}$ to obtain the *i*th feature:

(12) $$O^{i}={\mathrm{RF}}_{\delta_{s},\delta_{r}}\left(Q^{i}\right)$$

Here, RF represents the domain recursive filtering algorithm. $\delta_{s}$ is the filter’s spatial standard deviation, and $\delta_{r}$ is defined as the range standard deviation [60]. Then, we get the feature image $O=\left[O^{1},\cdots ,O^{N}\right]\in R^{M\times N}$.

Image fusion and recursive filtering (IFRF) is a simple yet powerful feature extraction method that aims at finding the best subset of hyperspectral bands that provide high classes’ separability. In other words, the IFRF feature method is used to select better bands that remove the noise and redundant information simultaneously [12]. Hence, we preprocess the HSIs firstly by IFRF to eliminate redundant information.

Let $X=O^{T}=\left[x_{1},x_{2},\cdots ,x_{M}\right]\in R^{N\times M}$ be the preprocessed features vector, where $x_{i}$ represents a pixel with N band numbers in the hyperspectral image, and *M* is the pixels number. We begin by sampling the *K* cluster centers and moving them to the lowest gradient position in the 3×3 neighborhood of the cluster centers. The squared Euclidean norm of the image is calculated by Equation (13):

(13) $$G(x,y)={∥I(x+1,y)-I\left(x-1,y\right)∥}^{2}+{∥I(x,y+1)-I\left(x,y-1\right)∥}^{2}$$

where I(x,y) represents the L,a,b vector corresponding to the pixel at position (x,y), and $∥.∥$ is the $l_{2}$-norm. This takes both color and intensity information into account.

Each pixel in the image is associated with the nearest cluster center whose search area overlaps the pixel. After each pixel is associated with its nearest cluster center, a new center is computed as the average Labxy vector of all the pixels in the cluster. Then, we iteratively associate the pixel with the nearest cluster center and recalculate the cluster center until convergence. We enforce the connection by relabeling the disjoint segmentations with their largest neighboring cluster’s label. The simple linear iterative clustering algorithm is summarized in Algorithm 1.

**Algorithm 1** SLIC superpixel segmentation.
**Input:** Processed HSIs image $X\in R^{N\times M}$, Desired number of approximately superpixels *K*.  1:Initialize cluster centers $C_{k}={\left[l_{k},a_{k},b_{k},x_{k},y_{k}\right]}^{T}$ by sampling pixels at regular grid steps *S*.  2:Move cluster centers to the lowest gradient position in a 3×3 neighborhood.  3:Set label l(i)=−1 for each pixel *i*.  4:Set distance d(i)=∞ for each pixel *i*.  5:
**Repeat**
  6:**for** each cluster center $C_{k}$ **do**  7:    **for** each pixel *i* in a 2S×2S region around $C_{k}$ **do**  8:        Compute the distance *D* between $C_{k}$ and *i*.  9:        **if** D<d(i) **then** 10:           set d(i)=D 11:           set l(i)=k 12:        **end if** 13:    **end for** 14:
**end for**
 15:Compute new cluster centers. 16:Compute residual error *E*. 17:**Until**E≤threshold. 18:Enforce connectivity.**Output:** Superpixel segmentations.


Hyperspectral images are segmented into many irregularly homogeneous regions. We stack the pixels in one homogeneous region into a matrix. We record the segment image as $X=\{X_{1},X_{2},\cdots ,X_{m}\}$, where k∈{1,2,⋯,m} is the index of the superpixels. *m* is the exact total number of superpixels in X. $n_{k}$ is the number of pixels in the superpixel $X_{k}$, and each column represents the bands of one pixel. Due to the low-rank attribute of the data, robust principal component analysis is used to extract the low-dimensional structure of all pixels in the homogeneous region. Then, the observed data matrix $X_{k}\in R^{N\times n_{k}}$ can be decomposed into a low-rank matrix and a sparse matrix. That is, $X_{k}=Z_{k}+E_{k}$, where $Z_{k}$ represents the low-rank matrix of $X_{k}$, and $E_{k}$ indicates the sparse noise matrix. Further, the RPCA optimization problem for each superpixel region is converted to the following form:

(14) $$\begin{array}{c} \min_{Z,E}\;{∥Z_{k}∥}_{*}+\lambda {∥E_{k}∥}_{1}\\ \;s.t.\;X_{k}=Z_{k}+E_{k} \end{array}$$

In the RPCA model, noise is required to be sparse, and the structural information of the noise is not considered. However, in machine learning or image processing fields, each column (or row) of the matrix has some meaning (e.g., a picture, and a signal data, etc.). Here, each column represents a pixel, which causes the noise to be sparse in column (or row). This structural information cannot be represented by the definition of an $l_{1}$-norm. To generate structural sparsity, the $l_{2,1}$-norm is proposed in the robust principal component analysis.

Unlike the $l_{1}$-norm, the $l_{2,1}$-norm produces sparsity based on columns (or rows). For the matrix $X\in R^{m\times n}$, its $l_{2,1}$- norm is [61,62]:

(15) $${∥X∥}_{2,1}=\sum_{j=1}^{n}\sqrt{\sum_{i=1}^{m}x_{i,j}^{2}}=\sum_{i=1}^{m}{∥X_{:,j}∥}_{2}$$

Thus, the $l_{2,1}$-norm is the sum of the $l_{2}$-norm of column vectors, which is also a measure of joint sparsity between vectors. For data that are interfered by structured noise, the following $l_{2,1}$-norm RPCA model in Equation (16) is very suitable:

(16) $$\begin{array}{c} \min_{Z,E}\;{∥Z_{k}∥}_{*}+\lambda {∥E_{k}∥}_{2,1}\\ \;s.t.\;X_{k}=Z_{k}+E_{k} \end{array}$$

In contrast to the original RPCA model, we call the error term with $l_{2,1}$-norm in Equation (16) as RPCA_2,1_. Here, the inexact augmented Lagrangian multiplier (ALM) algorithm [63,64] is utilized for the optimal solution $Z_{k}$.

The augmented Lagrangian multiplier method solves the optimization problem by minimizing the Lagrangian function as follows:

(17) $$\begin{array}{c} L\left(Z,E,Y,\mu \right)={∥Z∥}_{*}+\lambda {∥E∥}_{2,1}+\langle Y,X-Z-E\rangle +\frac{\mu }{2}{∥X-Z-E∥}_{F}^{2} \end{array}$$

where Y is defined as Lagrangian multipliers and μ>0 is the penalty parameter. The sub-problem for all variables is convex, which can supply a relevant and unique solution. Alternate iterations $Z_{k}$, $E_{k}$, and Y:

(18) $$\begin{array}{c} Z_{i+1}=\underset{Z}{\arg\;\min}L\left(Z,E_{i},Y_{i},\mu \right)\;\\ \;\;\;\;\;\;\;=\underset{Z}{\arg\;\min}{∥Z∥}_{*}+\langle Y_{i},X-Z-E_{i}\rangle +\frac{\mu }{2}{∥X-Z-E_{i}∥}_{F}^{2}\\ \;\;\;\;\;\;\;=\underset{Z}{\arg\;\min}{∥Z∥}_{*}+\frac{\mu }{2}{∥Z-(X-E_{i}+\frac{Y_{i}}{\mu })∥}_{F}^{2}\\ \;\;\;\;\;\;\;=\underset{Z}{\arg\;\min}\frac{1}{\mu }{∥Z∥}_{*}+\frac{1}{2}{∥Z-(X-E_{i}+\frac{Y_{i}}{\mu })∥}_{F}^{2} \end{array}$$

(19) $$\begin{array}{c} E_{i+1}=\underset{E}{\arg\;\min}L\left(Z_{i+1},E,Y_{i},\mu \right)\;\\ \;\;\;\;\;\;\;=\underset{E}{\arg\;\min}\lambda {∥E∥}_{2,1}+\langle Y_{i},X-Z-E_{i}\rangle +\frac{\mu }{2}{∥X-Z-E_{i}∥}_{F}^{2}\\ \;\;\;\;\;\;\;=\underset{E}{\arg\;\min}\lambda {∥E∥}_{2,1}+\frac{\mu }{2}{∥E-(X-Z+\frac{1}{\mu }Y)∥}_{F}^{2}\\ \;\;\;\;\;\;\;=\underset{E}{\arg\;\min}\frac{\lambda }{\mu }{∥E∥}_{2,1}+\frac{1}{2}{∥E-(X-Z+\frac{1}{\mu }Y)∥}_{F}^{2} \end{array}$$

Note that $Z_{i+1}$ and $E_{i+1}$ are convergent to ${Z_{i+1}}^{*}$ and ${E_{i+1}}^{*}$, respectively. Then, we update Y as follows:

(20) $$Y_{i+1}=Y_{i}+\mu_{i}\left(X-{Z_{i+1}}^{*}-{E_{i+1}}^{*}\right)\;$$

Finally, parameter μ is updated:

(21) $$\mu_{i+1}=\left\{\begin{array}{c} \rho \mu_{i},\;\;if\;\mu_{i}{∥{E_{i+1}}^{*}-{E_{i}}^{*}∥}_{F}/{∥X∥}_{F}<\varepsilon \;\\ \mu_{i},\;\;otherwise \end{array}\right.$$

where ρ>1 is a constant and ε>0 is a relatively small number.

The optimization process outline of the inexact ALM method [63] is given in Algorithm 2.

The initialization $Y_{0}=X/J\left(X\right)$ is defined as

(22) $$J\left(X\right)=\max\left({∥X∥}_{2},\lambda^{-1}{∥X∥}_{\infty }\right)$$

where ${∥\cdot ∥}_{\infty }$ is the maximum absolute value of the matrix entries.

**Algorithm 2** Inexact ALM method for solving RPCA.
**Input:** Observation matrix $X\in R^{m\times n}$ and parameter λ for local affinity.  1:Initialize: $Y_{0}=X/J\left(X\right);E_{0}=0;\mu_{0}>0;\rho >1;k=0$.  2:**while** not converged **do**  3:    //Lines 4-5 resolve $Z_{i+1}=\underset{Z}{\arg\;\min}L\left(Z,E_{i},Y_{i},\mu_{i}\right)$.  4:    $\left(U,S,V\right)=svd\left(X-E_{i}+{\mu_{i}}^{-1}Y_{i}\right)$;  5:    $Z_{i+1}=US_{{\mu_{i}}^{-1}}\left[S\right]V^{T}$.  6:    //Line 7 resolves $E_{i+1}=\underset{E}{\arg\;\min}L\left(Z_{i+1},E,Y_{i},\mu_{i}\right)$.  7:    $E_{i+1}=S_{{\mu_{i}}^{-1}}\left(X-Z_{i+1}+{\mu_{i}}^{-1}Y_{i}\right)$.  8:    $Y_{i+1}=Y_{i}+\mu_{i}\left(X-{Z_{i+1}}^{*}-{E_{i+1}}^{*}\right)\;;\mu_{i+1}=\rho \mu_{i}$.  9:    i←i+1. 10:
**end while**
**Output:** The low-rank matrix Z.


After RPCA_2,1_, we get the low-rank matrix $Z_{k}\in R^{N\times n_{k}}$ of each superpixel. Then, we merge these low-rank matrices to a whole low-rank graph $Z=\left\{Z_{1},Z_{2},\cdots ,Z_{m}\right\}=\left[z_{1},z_{2},\cdots ,z_{M}\right]\;\in \;R^{N\times M}$, which is the low-rank representation of $X=[x_{1},x_{2},\cdots ,x_{M}]$.

### 3.2. HSIs’ Classification Based on the SURPCA_2,1_ Graph

To overcome the “Curse of dimensionality” caused by the high dimension, we apply semi-supervised discriminant analysis to reduce the dimension. SDA is derived from linear discriminant analysis (LDA). The rejection matrix a can represent a priori consistency assumptions according to the regularization term [65]:

(23) $$a_{opt}=\underset{a}{\arg\;\max}\frac{a^{T}S_{b}a}{a^{T}S_{t}a+\alpha J\left(a\right)}$$

Here, $S_{t}$ is the total class scatter matrix. Parameter α is used to balance the complexity and empirical loss of the model. Empirical loss J(a) controls the learning complexity of the hypothesis family. The regularizer term J(a) incorporates the prior knowledge into some particular applications. When a set of unlabeled examples available, we aim to construct a J(a) incorporating the manifold structure.

Given a set of samples ${\left\{z_{i}\right\}}_{i=1}^{M}$, we can construct the graph G to represent the relationship between nearby samples by *k*NN. The samples $z_{i}$ and $z_{j}$ are considered as *k*-nearest neighbor if $z_{i}$ is among the *k*-nearest neighbor of $z_{j}$ or $z_{j}$ is among the *k*-nearest neighbor of $z_{i}$. Here, we employ the most simple 0–1 weighting [21] methods to assign weights for S:

(24) $$S_{ij}=\left\{\begin{array}{cc} 1, & if\;z_{i}\in N_{k}\left(z_{j}\right)\;or\;z_{j}\in N_{k}\left(z_{i}\right)\\ 0, & otherwise. \end{array}\right.$$

where $N_{k}\left(z_{i}\right)$ denotes *k*-nearest neighbor of $z_{i}$.

The SDA model incorporate the prior knowledge in the regularization term J(a), as follows:

(25) $$\begin{array}{c} J(a)=\sum_{ij}{\left(a^{T}z_{i}-a^{T}z_{j}\right)}^{2}S_{ij}\\ \;\;\;\;=2\sum_{i}a^{T}z_{i}D_{ii}{z_{i}}^{T}a-2\sum_{ij}a^{T}z_{i}S_{ij}z_{j}^{T}a\\ \;\;\;\;=2a^{T}Z\left(D-S\right)Z^{T}a\\ \;\;\;\;=2a^{T}\mathrm{ZL}Z^{T}a \end{array}$$

The diagonal matrix D, $D_{ii}=\sum_{j}S_{ij}$ is column (or row, since S is symmetric) sum of S. L=D−S is the Laplacian matrix [66]. Then, SDA objective function is:

(26) $$a_{opt}=\max_{a}\frac{a^{T}S_{b}a}{a^{T}\left(S_{t}+\alpha \mathrm{ZL}Z^{T}\right)a}$$

We obtain the projective vector a by maximizing the generalized eigenvalue problem where *d* is the weight matrix’s rank for the labeled graph.

(27) $$S_{b}a=\lambda \left(S_{t}+\alpha \mathrm{ZL}Z^{T}\right)a$$

The low-rank matrix extracted by RPCA captures the global correlation of the homogeneous region [37,39,67,68], whereas the *k*-nearest neighbor algorithm characterizes the local correlation of pixel points [69]. The probability that the neighboring pixels belong to the same category is large, which corresponds to the spatial distribution structure of the hyperspectral image. Therefore, the SURPCA_2,1_ graph can achieve good feature representations for graph-based semi-supervised dimensionality reduction.

Given a set of samples ${\left\{\left(z_{i},y_{i}\right)\right\}}_{i=1}^{l}$ and unlabeled samples ${\left\{z_{i}\right\}}_{i=l+1}^{m}$ with *c* classes, the $l_{k}$ is the *k*th class’s samples number. The algorithmic procedure of HSIs’ classification by applying the SLIC superpixel-based $l_{2,1}$-norm robust principal component analysis method is stated in the following paragraphs:

Step 1 Construct the adjacency graph: Construct the block low-rank and *k*NN graph S in Equation (24) for the regularization term. Furthermore, calculate the graph Laplacian L=D−S.

Step 2 Construct the labeled graph: For the labeled graph, construct the matrix as:

(28) $$W=\left[\begin{array}{cc} W_{l\times l} & 0\\ 0 & 0 \end{array}\right]$$

Define identity matrix $\tilde{I}=\left[\begin{array}{cc} I & 0\\ 0 & 0 \end{array}\right]\in R^{l\times l}$.

Step 3 Eigen-problem: Calculate the eigenvectors for the generalized eigenvector problem:

(29) $$\mathrm{ZW}Z^{T}a=\lambda Z\left(\tilde{I}+\alpha L\right)Z^{T}a$$

*d* is the rank of W, and $\left\{a_{1},a_{2},\cdots ,a_{d}\right\}$ is the *d* eigenvectors.

Step 4 Regularize discriminant analysis embedding: Let the transformation matrix $A=\left[a_{1},a_{2},\cdots ,a_{d}\right]\in R^{N\times d}$. Then, embedding the data into *d*-dimensional subspace,

(30) $$z\to z^{\prime }=A^{T}z$$

Step 5 Finally, apply the simple and ubiquitously-used classifiers nearest neighbor and Support Vector Machine (SVM) for classification.

**Figure 3:**
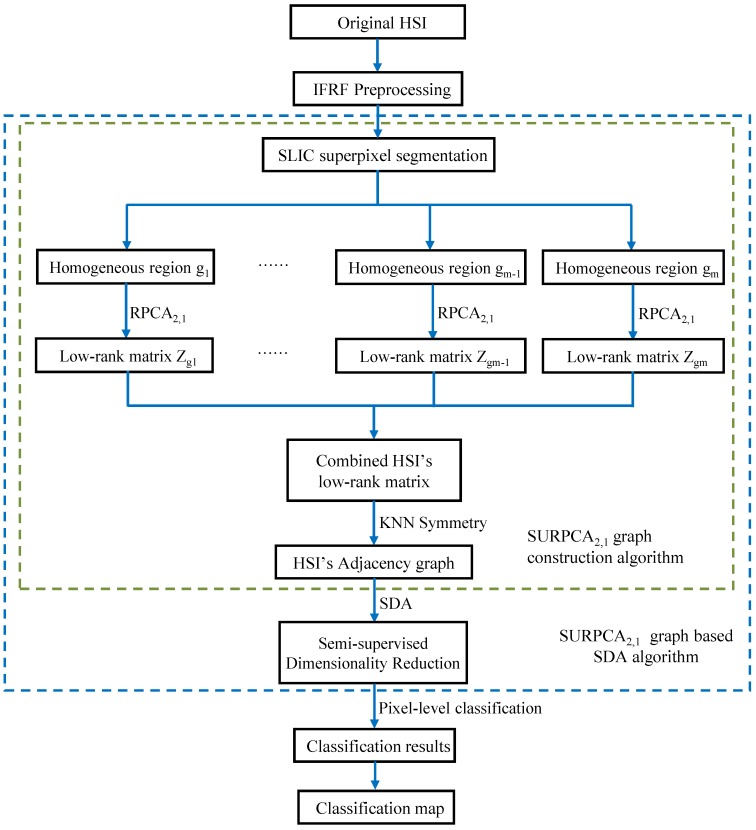
Model diagram of the SURPCA_2,1_ for hyperspectral image classification.

Figure 3 shows a model diagram of the SURPCA_2,1_ for hyperspectral image classification. The low-rank matrix measures the global correlation of the homogeneous regions, while the *k*NN algorithm preserves the local correlation of the pixels. The probability that adjacent pixels belong to the same category in the *k*NN is large, which corresponds to the spatial distribution structure of the hyperspectral image. Therefore, the SURPCA_2,1_ graph can be used to achieve high-quality data representation.

## 4. Experiments and Analysis

To investigate the performance of the SLIC superpixel-based $l_{2,1}$-norm robust principal component analysis graph model, we conducted extensive experiments on several real multi-class hyperspectral images. We performed experiments on a computer with CPU 2.60 GHz and 8 GB RAM.

### 4.1. Experimental Setup

#### 4.1.1. Hyperspectral Images

The Indian Pines (https://purr.purdue.edu/publications/1947/usage?v=1), Pavia University scene, and Salinas (http://www.ehu.eus/ccwintco/index.php/Hyperspectral_Remote_Sensing_Scenes) hyperspectral images were evaluated in the experiments.

**Figure 4:**
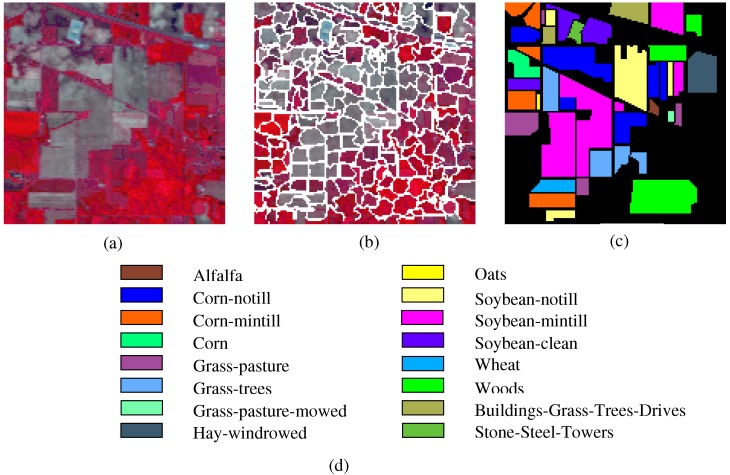
Indian Pines dataset: (**a**) false-color image; (**b**) segmentation map; and (**c**,**d**) ground truth image and reference data.

**Figure 5:**
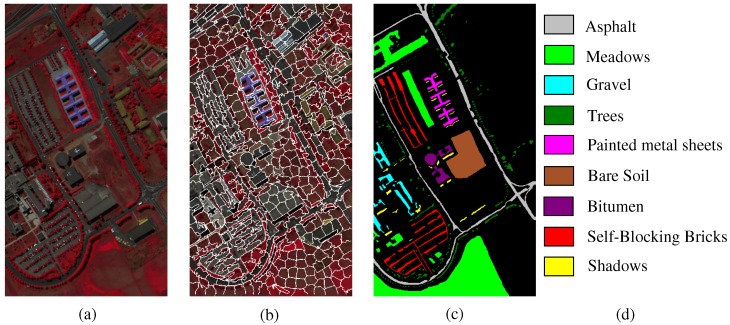
Pavia University scene; (**a**) false-color image; (**b**) segmentation map; and (**c**,**d**) ground truth image and reference data.

**Figure A1:**
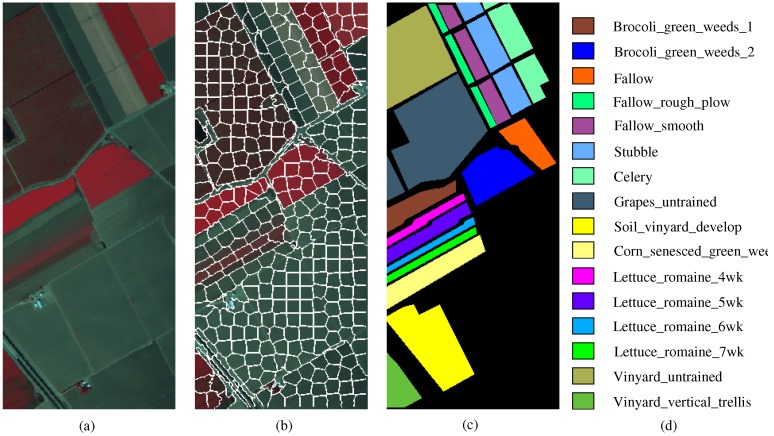
Salinas dataset: (**a**) false-color image; (**b**) segmentation map; and (**c**,**d**) ground truth image and reference data.

- The Indian Pines image is for the agricultural Indian Pine test site in Northwestern Indiana, which was a 220 Band AVIRIS Hyperspectral Image Data Set: 12 June 1992 Indian Pine Test Site 3. It was acquired over the Purdue University Agronomy farm northwest of West Lafayette and the surrounding area. The data were acquired to support soils research being conducted by Prof. Marion Baumgardner and his students [70]. The wavelength is from 400 to 2500 nm. The resolution is 145 × 145 pixels. Because some of the crops present (e.g., corn and soybean) are in the early stages of growth in June, the coverage is minuscule—approximately less than 5%. The ground truth is divided into sixteen classes, and are not all mutually exclusive. The Indian Pines false-color image and the ground truth image are presented in Figure 4.
- The Pavia University scene was obtained from an urban area surrounding the University of Pavia, Italy on 8 July 2002 with a spatial resolution of 1.3 m. The wavelength ranges from 0.43 to 0.86 μm. There are 115 bands with size 610 × 340 pixels in the image. After removing 12 water absorption bands, 103 channels were left for testing. The Pavia University scene’s false-color image and the corresponding ground truth image are shown in Figure 5.

- The Salinas image is from the Salinas Valley, California, USA, which was obtained from an AVIRIS sensor with 3.7 m spatial resolution. The image includes a size of 512 × 217 with 224 bands. Twenty water absorption bands were discarded here. There are 16 classes containing vegetables, bare soil, vineyards, etc. Figure A1 displays the Salinas image’s false-color and ground truth images.

#### 4.1.2. Evaluation Criteria

We give some evaluation criteria to evaluate the proposed method for HSIs, as follows.

Classification accuracy (CA) is the classification accuracy of each category in the image. The confusion matrix [71] is often used in the remote sensing classification field, and its form is defined as: $M={\left[m_{ij}\right]}_{n\times n}$, where $m_{ij}$ indicates that the number of pixels labeled by the *j* class should belong to the *i* class. *n* is the class number. The reliability of classification depends on the diagonal values of the confusion matrix. The higher the values on the diagonal of the confusion matrix are, the better the classification results will be.

We also used the following three main indicators: overall accuracy (OA), average accuracy (AA), and the kappa coefficient [72]. OA refers to the percentage of the overall correct classification. AA estimates the average correct classification percentage for all categories. The kappa coefficient takes the chance agreement into account and fixes it, whereas OA and AA check how many pixels are classified correctly. Assuming that the reference classification (i.e., ground truth) is true, then how well they agree is measured. Here, we assumed that both classification and reference classification were independent class assignments of equal reliability. The advantage of the kappa coefficient over overall accuracy is that the kappa coefficient considers chance agreement and corrects it. The chance agreement is the probability that the classification and reference classification agree by mere chance. Assuming statistical independence, we obtained this probability estimation [73,74].

#### 4.1.3. Comparative Algorithms

We give several comparative methods to illustrate the great improvement by the SLIC superpixel-based $l_{2,1}$-norm robust principal component analysis graph model in the HSIs’ classification. For fairness, these comparative algorithms incorporate the simple linear iterative clustering (SLIC), which are shown below.

- RPCA (robust principal component analysis) method [63]: The original robust principal component analysis with $l_{1}$-norm.
- PCA (principal component analysis) method [63]: PCA [16] seeks the best low-rank representation of the given data matrix that minimizes the difference between Z and X:
  (31) $$\begin{array}{c} \min_{Z,E}\;{∥E∥}_{F}\\ \;s.t.\;\;rank(Z)\leq r,X=Z+E \end{array}$$
- IFRF (image fusion and recursive filtering) algorithm.
- Origin: Original bands of the unprocessed image.

### 4.2. Classification of Hyperspectral Images

We performed experiments on the three hyperspectral images to examine the performance of the SURPCA_2,1_ graph. Ten independent runs of each algorithm were evaluated by resampling the training samples in each run. We chose the mean values as the results. In practice, it is difficult to obtain labeled samples, while unlabeled samples are often available and in large numbers. Unlike most of the existing HSI classifications, we tested the performance of all comparative methods using only small rate of the labeled samples. Table 1 shows the training and testing sets for all datasets, where the training sets are chosen randomly. The training samples were approximately 4%, 3%, and 0.4% for the three images, which were minimal sets to the entire dataset. Considering the classes with a meager number of samples, we incorporated a minimum threshold of training samples. Here, we set the minimum threshold of training samples for each class as five, which can eradicate the difference between the classes with a low number of samples. The SLIC superpixels’ number for the three HSIs is 200, 600, and 400, respectively. Figure 4b, Figure 5b and Figure A1b show the segmentation maps. The amount of reduced dimension is 30 for the three hyperspectral images. The spatial standard deviation and range standard deviation of the filter are $\delta_{s}$ and $\delta_{r}$ with 200 and 0.3, respectively. The parameter σ in *k*-nearest neighbor $S_{ij}=\exp(-\frac{{∥z_{i}-z_{j}∥}^{2}}{2\sigma^{2}})$ is 0.1, which is provided randomly.

**Figure 6:**
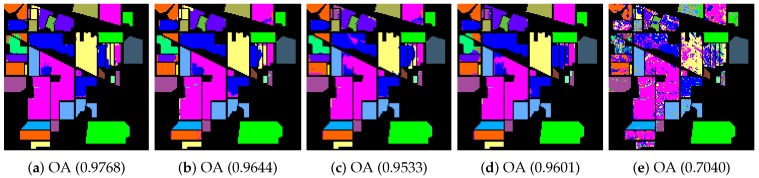
Indian Pines image classification results: (**a**) robust principal component analysis (RPCA_2,1_); (**b**) RPCA; (**c**) PCA; (**d**) image fusion and recursive filtering (IFRF); and (**e**) Origin. OA, overall accuracy.

**Figure 7:**
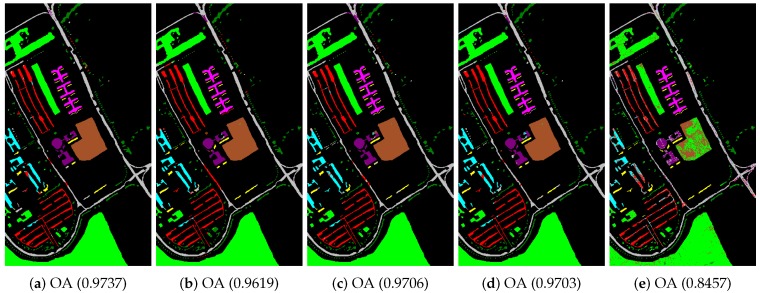
Pavia University scene classification results: (**a**) RPCA_2,1_; (**b**) RPCA; (**c**) PCA; (**d**) IFRF; and (**e**) Origin. OA, overall accuracy.

**Figure A2:**
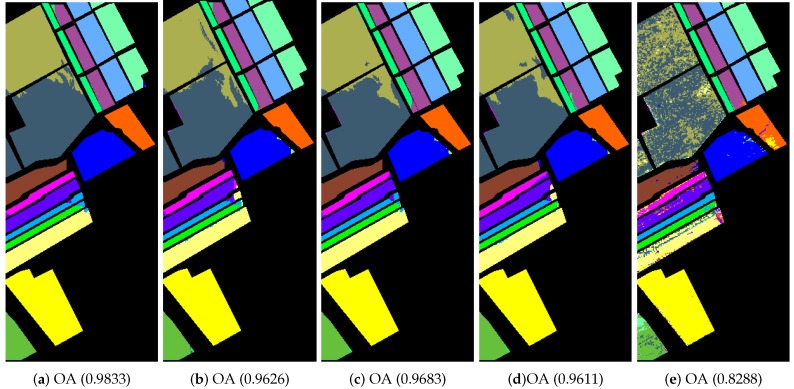
Salinas image Classification results: (**a**) RPCA_2,1_; (**b**) RPCA; (**c**) PCA; (**d**) IFRF; and (**e**) Origin. OA, overall accuracy.

In the beginning, we utilized different manifold mapping graphs to obtain the manifold matrix of the hyperspectral images. Then, we applied the SDA algorithm for semi-supervised dimensionality reduction. Both the nearest neighbor and support vector machine algorithms were applied as the classifiers here to test and verify the proposed SURPCA_2,1_ graph. Table 2, Table 3 and Table A1 give detailed classification results for CA, OA, AA, and kappa coefficients received from different methods, where bold numbers represent the best ones for various graphs. Figure 6, Figure 7 and Figure A2 indicate classification maps of the hyperspectral images acquired by several methods (randomly selected from our experiments above) with the NN classifier. It was observed that the results in the figures were random for each method. We analyzed the running times of different models on the Indian Pines image, Pavia University scene, and Salinas scene image. Ten separate runs were calculated for the total time.

In Table 2, Table 3, and Table A1, we can notice that the SURPCA_2,1_ graph is superior to the other graph models. Therefore, it significantly improves the classification performance of hyperspectral images, which indicates that the SURPCA_2,1_ graph is a superior HSIs feature extraction method with both NN and SVM classifiers. For example, in Table 2, although the overall accuracy and kappa coefficient of other graphs are high, the average accuracy is not good. The accuracy of Grass-pasture-mowed and Oats are more (i.e., 12–37%) improved with the NN classifier and with SVM classifier (i.e., 25–64%) than the compared graphs. In addition, the Corn, Soybean-notill, and Soybean-clean classes are significantly improved, especially the Grass-pasture-mowed, for the Indian Pines image. For the Salinas dataset, the classification of Fallow, Lettuce_romaine_6wk, and Vinyard_untrained classes are obviously improved. We can see that, although sometimes the overall accuracy is not very different, the average accuracy is far ahead of the compared graphs. This is because, when there are fewer samples in some classes, it is very easy to be confused. For the Indian Pines image, the accuracy of all categories is more than 89.81%, except for the 81.96% accuracy of the Grass-pasture-mowed with the NN classifier, and it is also more than 95.03% with the SVM classifier, which meets the general accuracy requirement of remote sensing. For the Pavia University scene and Salinas image, the accuracy of all categories is larger than 91.69% and 91.95%, and 91.7% and 97.45% with NN and SVM, respectively. We can also see that our graph performed well in all categories with both of the classifiers. However, for other methods, they might perform much worse in some confusing categories, especially with the NN classifier. This phenomenon shows that our proposed graph is robust to classifier methods.

We analyzed the running times of different models on the three hyperspectral images. We used ten separate runs to calculate the total time. We give the mean running time. As shown in Table 4, the execution time of the SURPCA_2,1_ graph method is slightly longer than the other methods. Although our algorithm is slower than the traditional algorithms, the performance is much better than these baselines at an acceptable running time. Note that the RPCA_2,1_ is significantly faster than the classic RPCA. This is owing to the superior ability of the $l_{2,1}$-norm to converge more quickly than the $l_{1}$-norm.

Although the classification results with NN are slightly worse with the SVM algorithm, the running time is much less than the SVM classifier. Moreover, in Table 2, Table 3, and Table A1, we can see that the classification results with NN classifier are acceptable. Therefore, in the following experiments, the nearest neighbor is applied.

### 4.3. Robustness of the SURPCA_2,1_ Graph

We evaluated the performance of the proposed SURPCA_2,1_ graph. To fully determine the superiority of the proposed graph model, we also analyzed the robustness of SURPCA_2,1_. Considering the practical situation, we analyzed the robustness of SURPCA_2,1_ on the size of the labeled samples and noise situation.

#### 4.3.1. Labeled Size Robustness

**Figure 8:**
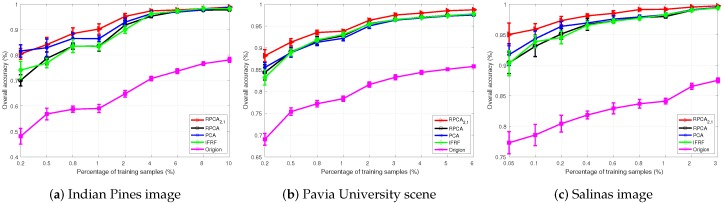
HSIs classification accuracy with varying labeled ratio.

We analyzed the impact of different sizes of training and testing sets. The experiments were carried out with ten independent runs for each algorithm. We calculated the mean values and standard deviations of the results. Figure 8 shows the classification results of different graph models. It compares the overall classification results with different training size samples in each class. The training samples percentages were 0.2–10%, 0.2–6%, and 0.05–3% for Indian Pines image, Pavia University scene, and Salinas image, respectively.

In most cases, our graph model SURPCA_2,1_ consistently achieves the best results, which are robust to the label percentage variations. With increasing size of the training sample, OA generally increases in all methods, showing a similar trend. When the labeled ratio is fixed, the SURPCA_2,1_ method is usually superior to others. Similarly, the three classification criteria are increased with the increasing training samples simultaneously.

Note that, our proposed graph realize higher classification results, even with a low label ratio. The other comparison algorithms are especially not as robust as the SURPCA_2,1_ graph with low label ratio. In particular, we can see that our graph model perform relatively better, and the difference between other comparison methods is larger when the labeling rate is smaller. Therefore, due to the high cost and difficulty of labeled samples, our proposed graph is much more robust and suitable for real-world hyperspectral images.

#### 4.3.2. Noise Robustness

**Figure 9:**
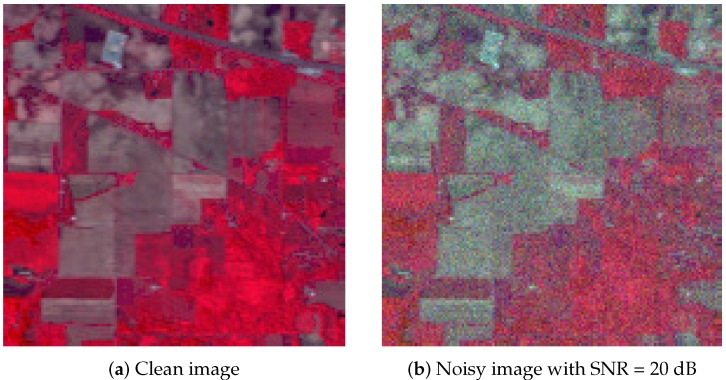
False-color clean and noisy images of the Indian Pines image.

We evaluated the robustness to noise of the SURPCA_2,1_ graph on the three hyperspectral images. We added zero-mean Gaussian noise with a signal-to-noise ratio (SNR) of 20 dB to each band. Figure 9 shows an example noise image of three randomly selected bands in the Indian Pines image, which is similar to the other two hyperspectral images.

**Figure 10:**
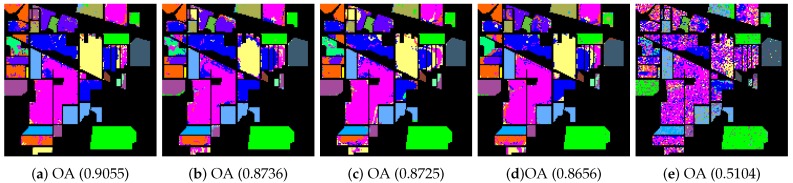
Noisy Indian Pines image classification results: (**a**) RPCA_2,1_; (**b**) RPCA; (**c**) PCA; (**d**) IFRF; and (**e**) Origin. OA, overall accuracy.

**Figure 11:**
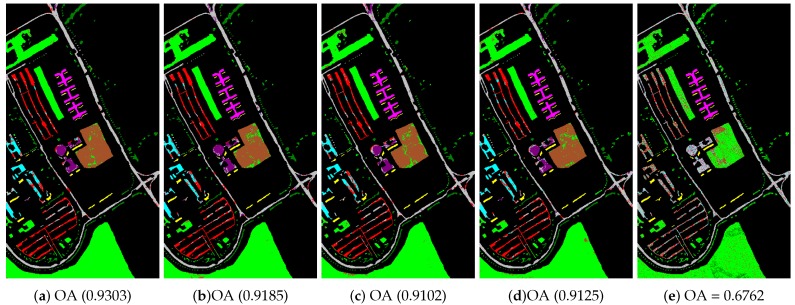
Noisy Pavia University scene classification results: (**a**) RPCA_2,1_; (**b**) RPCA; (**c**) PCA; (**d**) IFRF; and (**e**) Origin. OA, overall accuracy.

**Figure A3:**
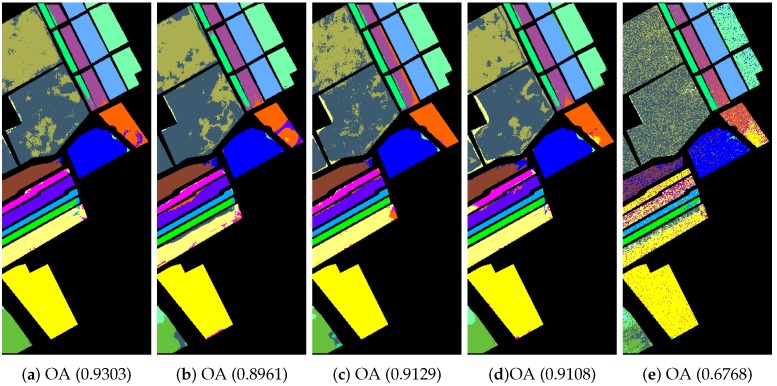
Noisy Salinas image classification results: (**a**) RPCA_2,1_; (**b**) RPCA; (**c**) PCA; (**d**) IFRF; and (**e**) Origin. OA, overall accuracy.

We evaluated different graphs’ performance in the noisy situation. Each algorithm ran ten times independently, with re-sampling of training samples. We calculated the mean and standard deviation results. The labeled sampling ratios were 4%, 3%, and 0.4%, respectively, as described in Table 5. The bold numbers represent the best results for various graphs. Figure 10, Figure 11, and Figure A3 display classification maps of the three noise hyperspectral images correlated with OA values (randomly selected from our experiments above). It is observed that the results in the figures were random for each method.

From the results of these different graph models, we can see that our graph is robust to noise and the sample size of the labeled set. In a noisy environment, our method is relatively less degraded and more robust. With few labeled samples, the SURPCA_2,1_ graph is more forceful than the other graphs due to the $l_{2,1}$-norm robust principal component analysis noise robustness. Moreover, the SURPCA_2,1_ graph performs very well for all three experimental hyperspectral images.

### 4.4. Parameters of SURPCA_2,1_ Model

**Figure 12:**
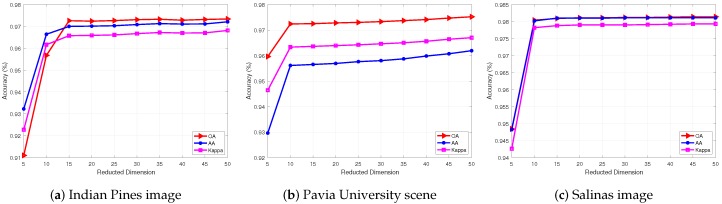
Classification accuracy of HSIs with different reduced dimensions.

We evaluated the SURPCA_2,1_ graph’s parameters superpixel number *m* and the reduced dimensions of SDA. We conducted ten independent runs per algorithm and calculated the mean values and standard deviations of the results. Table 6 and Figure 12 show the performance of different superpixel number *m* and the reduced dimensions, respectively. For these three hyperspectral images, the labeled ratio was about 4%, 3%, and 0.4%, respectively. The superpixel segmentation numbers are shown in Table 6 for the three hyperspectral images.

In general, the classification results are not greatly affected by the number of superpixel segmentations, indicating that our graph is robust to the number of superpixel segmentations. It is observed that the increase in superpixel numbers simultaneously accelerated the running time considerably. However, the impact is relatively small when the number of samples is large. Therefore, we could use relatively small superpixel numbers in actual situations for the purpose of efficiency with minimal overall loss of classification accuracy.

We can observe that the reduction dimension is robust in Figure 12. It will reach steady high accuracy at a relatively low dimension such as 10 or 15.

## 5. Discussion

The hyperspectral images’ classification plays a pivotal role in HSIs’ application. In the present work, we propose a novel graph construction model for HSIs’ semi-supervised learning, SLIC superpixel-based $l_{2,1}$-norm robust principal component analysis. Our goal is to improve HSIs’ classification results through useful graph model. The experimental results show that SURPCA_2,1_ is a competitive graph model for hyperspectral images’ classification.

Table 2, Table 3, and Table A1 show that SURPCA_2,1_ is an effective graph model that achieves the highest classification accuracy. Even with simple classifiers and few labeled samples, the performance is excellent. In most cases, the graph model SURPCA_2,1_ obtains the highest classification results, which indicates that the SURPCA_2,1_ graph is a good graph construction model and can notably enhance the hyperspectral images’ classification performance. In some cases, other graph models (e.g., RPCA and PCA) may perform well in some categories, but they are not as robust as our graph in all categories. This is because the structural information cannot be well represented from the definition of $l_{1}$-norm, while the $l_{2,1}$-norm RPCA_2,1_ produces sparsity based on columns (or rows). The SLIC superpixel segmentation method considers the spatial correlation of pixels in hyperspectral images. For an HSI, the correlation of the adjacent pixels is usually very high since their spectral features are approximately in the same subspace [41]. By extracting a low-rank matrix in each homogeneous region, we can reveal the low-dimensional structure of pixels and enhance the classification results. Consequently, the SURPCA_2,1_ graph model is a superior hyperspectral image’s feature graph which significantly improves the performance of HSIs’ classification.

We analyzed the robustness to variations in the labeled samples ratio and noisy situations. Figure 8 shows that the SURPCA_2,1_ graph model provides the best results, which are robust to the varying label percentage. Figure 8 also shows that the proposed graph achieves high classification accuracy even at meager label rates, while other graphs may not be as robust as the SURPCA_2,1_ graph, particularly when the label rate is low. Therefore, since labeling data is a costly and difficult task, our SURPCA_2,1_ model is more suitable and robust for real-world applications. From these results of different graph models, we can see that our graph is robust to the sample ratio of the labeled set as well as the noisy environments. Upon adding noise, the performance of the SURPCA_2,1_ graph does not see a large decrease. However, for the comparative graphs, the overall accuracy drops much more than that of SURPCA_2,1_. In addition to the India Pines image, we could see the same results from the other two hyperspectral images. This benefits from the robustness of the $l_{2,1}$-norm robust principal component analysis to sparse noise, whereas RPCA and PCA may be sensitive to sparse noise. Therefore, the SURPCA_2,1_ graph is robust to noisy environments and the labeled sample ratio, which indicates that it is highly competitive in practical situations.

We evaluated the performance of parameters with different superpixel segmentations number and reduction dimensions for the SDA method. In Table 6, we can see that the classification results are not greatly affected by the number of superpixel segmentations, indicating that our graph is robust to the number of superpixel segmentations. Therefore, we could use a relatively small number of superpixels in actual situations for the purpose of efficiency, with barely any classification accuracy loss. Moreover, in Figure 12, we can see that the classification results are robust after the dimension reached a certain numerical value. It reaches a high accuracy at a relatively low dimension (e.g., 10 or 15). Overall, our proposed SURPCA_2,1_ graph is very much robust and shows excellent performance in the classification of HSIs.

## 6. Conclusions

In this paper, we present a novel graph for HSI feature extraction, referred to as SLIC superpixel-based $l_{2,1}$-norm based robust principal component analysis. The SLIC superpixel segmentation method considers the spatial correlation of pixels in HSIs. The $l_{2,1}$-norm robust principal component analysis is used to extract the low-rank structure of all pixels in each homogeneous area, which captures the spectral correlation of the hyperspectral images. Therefore, the classification performance of the SURPCA_2,1_ graph is much better than the compared methods RPCA, PCA, etc. Experiments on real-world HSIs indicated that the SURPCA_2,1_ graph is a robust and efficient graph construction model.

## Figures and Tables

**Table 1:** Training and testing samples for the three hyperspectral images.

Class	Indian Pines Image			Pavia University Scene			Salinas Image		
	Train	Test	Sample No.	Train	Test	Sample No.	Train	Test	Sample No.
C1	7	39	46	205	6426	6631	14	1995	2009
C2	63	1365	1428	565	18,084	18,649	20	3706	3726
C3	39	791	830	69	2030	2099	13	1963	1976
C4	15	222	237	97	2967	3064	11	1383	1394
C5	25	458	483	46	1299	1345	16	2662	2678
C6	35	695	730	156	4873	5029	21	3938	3959
C7	7	21	28	45	1285	1330	20	3559	3579
C8	25	453	478	116	3566	3682	51	11,220	11,271
C9	6	14	20	34	913	947	30	6173	6203
C10	44	928	972	-	-	-	19	3259	3278
C11	104	2351	2455	-	-	-	10	1058	1068
C12	29	564	593	-	-	-	13	1914	1927
C13	14	191	205	-	-	-	9	907	916
C14	56	1209	1265	-	-	-	10	1060	1070
C15	21	365	386	-	-	-	35	7233	7268
C16	9	84	93	-	-	-	13	1794	1807
Total	702	9547	10,249	1762	41,014	42,776	305	53,824	54,129

**Table 2:** Indian Pines image classification results.

Method	RPCA_2,1_	RPCA	PCA	IFRF	Origion	RPCA_2,1_	RPCA	PCA	IFRF	Origin
**Classifier**	**NN**					**SVM**				
C1	**1**	**1**	0.9870	0.9809	0.8051	**1**	**1**	**1**	**1**	0.9473
C2	0.9686	0.9442	0.9380	0.9487	0.4961	0.9676	**0.9701**	0.9295	0.9482	0.5169
C3	0.9667	0.9524	**0.9718**	0.9342	0.6572	0.9659	0.9679	0.9426	0.9574	0.3793
C4	0.9578	0.9698	0.9589	0.9122	0.3964	**0.9819**	0.9133	0.9383	0.9420	0.5671
C5	0.9868	0.9463	0.9384	0.9687	0.8225	**0.9933**	0.9856	0.9821	0.9868	0.8809
C6	0.9837	0.9833	0.9394	0.9483	0.7568	**1**	**1**	0.9986	0.9986	0.8476
C7	0.8686	0.7254	0.6164	0.8107	0.6786	**1**	0.9375	**1**	0.9886	0.9188
C8	**1**	**1**	**1**	**1**	0.9486	**1**	**1**	**1**	**1**	0.9367
C9	0.9800	0.7812	0.8496	0.7469	0.5042	**1**	**1**	**1**	**1**	0.8042
C10	0.9342	0.9139	0.9215	0.8742	0.6777	0.9647	0.9618	**0.9743**	0.9471	0.4916
C11	0.9721	0.9700	0.9748	**0.9749**	0.6933	0.9672	0.9604	0.9626	0.9677	0.5560
C12	0.9602	0.9024	0.9753	0.9344	0.6833	0.9854	**0.9908**	0.9658	0.9701	0.6454
C13	0.9705	**1**	0.9748	0.9935	0.9240	**1**	**1**	**1**	0.9987	0.9909
C14	0.9952	0.9895	0.9939	0.9915	0.9217	0.9954	**0.9996**	0.9975	0.9975	0.9190
C15	0.9624	0.9711	0.9768	0.9379	0.6666	0.9916	0.9937	**0.9958**	0.9897	0.7551
C16	**0.9905**	0.9881	0.9881	0.9881	0.9871	0.9866	0.9859	0.9712	0.9846	0.9825
OA	0.9706	0.9585	0.9594	0.9514	0.7077	**0.9786**	0.9759	0.9682	0.9713	0.6536
AA	0.9686	0.9399	0.9378	0.9341	0.7262	**0.9875**	0.9792	0.9787	0.9798	0.7587
Kappa	0.9664	0.9527	0.9536	0.9446	0.6633	**0.9755**	0.9724	0.9636	0.9672	0.6000

**Table 3:** Pavia University scene classification results.

Method	RPCA_2,1_	RPCA	PCA	IFRF	Origion	RPCA_2,1_	RPCA	PCA	IFRF	Origin
**Classifier**	**NN**					**SVM**				
C1	0.9581	0.9630	0.9632	0.9581	0.8969	**0.9771**	0.9745	0.9698	0.9657	0.8119
C2	0.9925	0.9942	0.9935	0.9944	0.8491	0.9957	**0.996**	0.9956	0.9951	0.8676
C3	0.9465	0.9420	0.9212	0.9543	0.6836	**0.9758**	0.9678	0.9715	0.9578	0.6425
C4	0.9923	0.9841	0.9865	0.9880	0.9360	0.9918	0.9889	**0.9938**	0.9895	0.8802
C5	**0.9993**	0.9989	0.9957	0.9985	0.9929	0.917	0.9377	0.9433	0.9355	0.9972
C6	0.9966	0.9953	0.9921	**0.9981**	0.7228	0.9965	0.9963	0.9953	0.9977	0.7286
C7	0.9177	0.9090	0.8921	0.8822	0.7385	**0.9751**	0.9689	0.9642	0.963	0.6193
C8	0.9169	0.9082	0.9182	0.9135	0.7990	**0.9656**	0.9589	0.9575	0.9574	0.7057
C9	0.9353	0.9302	0.9369	0.9509	**1**	0.964	0.9577	0.9546	0.9575	0.9996
OA	0.9751	0.9744	0.9734	0.9748	0.8425	**0.9849**	0.9839	0.9832	0.9815	0.8238
AA	0.9617	0.9583	0.9555	0.9598	0.8465	**0.9732**	0.9719	0.9717	0.9688	0.8058
Kappa	0.9670	0.9660	0.9647	0.9666	0.7869	**0.98**	0.9787	0.9777	0.9754	0.7628

**Table 4:** Different graphs’ running time on real-word hyperspectral images (HSIs) (unit:s). NN, nearest neighbor; SVM, support vector machine.

Method	RPCA_2,1_	RPCA	PCA	IFRF	Origion	RPCA_2,1_	RPCA	PCA	IFRF	Origin
**Classifier**	**NN**					**SVM**				
Indian Pines image	16.73	21.62	5.16	5.31	5.69	27.94	34.53	14.39	16.90	17.34
University of Pavia	83.89	100.31	54.28	56.92	47.11	115.84	135.86	88.52	90.26	116.61
Salinas image	15.55	18.14	4.24	4.27	3.22	28.56	32.61	14.39	14.32	15.29

**Table 5:** Classification results with Gaussian noise on three HSIs.

Images		RPCA_2,1_	RPCA	PCA	IFRF	Origin
Indian Pines image	OA	0.9004 ± 0.0103	0.8615 ± 0.0137	0.8736 ± 0.0067	0.86261 ± 0.0937	0.5008 ± 0.006
	AA	0.9198 ± 0.0179	0.8481 ± 0.0261	0.8573 ± 0.024	0.8367 ± 0.0231	0.4530 ± 0.0256
	Kappa	0.8863 ± 0.011	0.8426 ± 0.0143	0.8561 ± 0.0257	0.8434 ± 0.00763	0.4230 ± 0.0069
University of Pavia image	OA	0.9261 ± 0.0041	0.9156 ± 0.004	0.9116 ± 0.0057	0.9182 ± 0.0035	0.6721 ± 0.0079
	AA	0.9212 ± 0.0054	0.9085 ± 0.0066	0.9055 ± 0.0107	0.9105 ± 0.0077	0.6162 ± 0.0088
	Kappa	0.9012 ± 0.0055	0.8871 ± 0.0055	0.8815 ± 0.0078	0.8906 ± 0.0048	0.5527 ± 0.0097
Salinas image	OA	0.9329 ± 0.0078	0.911 ± 0.0062	0.9125 ± 0.0072	0.9095 ± 0.0108	0.6658 ± 0.0101
	AA	0.9523 ± 0.0051	0.9268 ± 0.0054	0.9294 ± 0.0089	0.9247 ± 0.0102	0.7088 ± 0.0098
	Kappa	0.9253 ± 0.0086	0.901 ± 0.0069	0.9027 ± 0.008	0.8994 ± 0.012	0.625 ± 0.0106

**Table 6:** SURPCA_2,1_ graph’s classification accuracy with different segmentation numbers.

Images	No. *m* of Superpixels	OA	AA	Kappa	Time (s)
Indian Pines image	104	0.9633 ± 0.0072	0.9507 ± 0.027	0.9581 ± 0.0091	12.43
	226	0.9703 ± 0.006	0.9687 ± 0.032	0.9661 ± 0.0062	17.96
	329	0.9721 ± 0.0053	0.9657 ± 0.0254	0.9682 ± 0.0069	21.80
University of Pavia image	195	0.9697 ± 0.0035	0.9473 ± 0.0061	0.9598 ± 0.0047	75.15
	386	0.9711 ± 0.0043	0.9564 ± 0.0084	0.9616 ± 0.0058	78.33
	594	0.9753 ± 0.0026	0.9614 ± 0.0046	0.9672 ± 0.0035	81.20
	813	0.975 ± 0.0018	0.9596 ± 0.0053	0.9668 ± 0.0024	85.97
Salinas image	187	0.9666 ± 0.0059	0.9743 ± 0.0064	0.9629 ± 0.0065	87.71
	389	0.978 ± 0.0042	0.9813 ± 0.003	0.9755 ± 0.0047	92.96
	593	0.9823 ± 0.0037	0.9826 ± 0.003	0.9804 ± 0.0041	98.91

## Appendix A

**Table A1:** Salinas image classification results.

Method	RPCA_2,1_	RPCA	PCA	IFRF	Origion	RPCA_2,1_	RPCA	PCA	IFRF	Origin
**Classifier**	**NN**					**SVM**				
C1	**1**	0.9999	**1**	0.932	0.9726	**1**	**1**	**1**	**1**	0.9982
C2	**0.9994**	0.9929	0.9952	0.9651	0.9796	0.9926	0.9959	0.9969	0.9789	0.9913
C3	0.9969	0.997	0.9949	0.9501	0.8262	**0.9990**	0.9984	0.9986	0.9982	0.9652
C4	0.9633	0.916	0.9081	0.8496	0.9703	**0.9745**	0.9718	0.9715	0.9542	0.9685
C5	0.9903	0.966	0.983	0.9592	0.9581	**0.9997**	0.9991	0.9986	0.9990	0.9001
C6	**1**	**1**	**1**	0.9952	0.9958	0.9997	0.9997	0.9998	0.9916	0.9997
C7	0.9971	0.9881	0.9901	0.9692	0.9202	**0.9991**	0.9975	0.9986	0.9900	0.9989
C8	0.9882	0.9666	0.9714	0.8995	0.6584	**0.9958**	0.9925	0.9927	0.9881	0.7304
C9	**0.9999**	0.9947	0.9976	0.9932	0.9408	0.9998	0.9995	0.9995	0.9979	0.9592
C10	**0.9969**	0.9905	0.9946	0.9681	0.8393	0.9957	0.9888	0.9931	0.9886	0.9283
C11	0.9961	0.982	0.9926	0.9548	0.851	**1**	0.9994	0.9989	0.9866	0.951
C12	0.9563	0.9481	0.9631	0.9259	0.8886	**0.9907**	0.9786	0.9819	0.9903	0.8949
C13	0.9394	0.8897	0.9354	0.7101	0.763	**0.9999**	0.9982	0.993	0.9406	0.8367
C14	**0.9865**	0.9845	0.9725	0.9791	0.9145	0.9759	0.9835	0.9688	0.9837	0.864
C15	0.9195	0.8994	0.8874	0.7298	0.5459	0.9980	0.9966	**0.9983**	0.9771	0.6202
C16	**1**	0.9998	0.9994	0.9944	0.9398	0.9996	0.9993	0.9999	0.9911	0.9955
OA	0.9809	0.9668	0.9692	0.9653	0.8185	**0.9964**	0.9947	0.9951	0.9867	0.866
AA	0.9831	0.9697	0.9741	0.9705	0.8727	**0.995**	0.9937	0.9931	0.9847	0.9126
Kappa	0.9787	0.963	0.9657	0.9614	0.7976	**0.996**	0.9941	0.9945	0.9852	0.8506
